# Flavonoids in Treatment of Chronic Kidney Disease

**DOI:** 10.3390/molecules27072365

**Published:** 2022-04-06

**Authors:** Yi-Ling Cao, Ji-Hong Lin, Hans-Peter Hammes, Chun Zhang

**Affiliations:** 1 Department of Nephrology, Union Hospital, Tongji Medical College, Huazhong University of Science and Technology, Wuhan 430022, China; saintiker@foxmail.com; 2 5th Medical Department, Medical Faculty Mannheim, University of Heidelberg, D-68167 Mannheim, Germany; jihong.lin@medma.uni-heidelberg.de (J.-H.L.); hp.hammes@umm.de (H.-P.H.)

**Keywords:** chronic kidney disease, flavonoids, oxidative stress, inflammation, nephroprotection

## Abstract

Chronic kidney disease (CKD) is a progressive systemic disease, which changes the function and structure of the kidneys irreversibly over months or years. The final common pathological manifestation of chronic kidney disease is renal fibrosis and is characterized by glomerulosclerosis, tubular atrophy, and interstitial fibrosis. In recent years, numerous studies have reported the therapeutic benefits of natural products against modern diseases. Substantial attention has been focused on the biological role of polyphenols, in particular flavonoids, presenting broadly in plants and diets, referring to thousands of plant compounds with a common basic structure. Evidence-based pharmacological data have shown that flavonoids play an important role in preventing and managing CKD and renal fibrosis. These compounds can prevent renal dysfunction and improve renal function by blocking or suppressing deleterious pathways such as oxidative stress and inflammation. In this review, we summarize the function and beneficial properties of common flavonoids for the treatment of CKD and the relative risk factors of CKD.

## 1. Introduction

For thousands of years, natural products have been widely used in many regions of the world. These products have a wide range of biological activities and can be found in almost all fruits, flowers, seeds, vegetables, and minerals. Currently, with the rapid development of technology, natural products have gained increasing popularity in many Western countries. Extensive experience and clinical application of many natural products have been accumulated and combined with continuous improvements in chemical technologies and biological methods to treat diseases with little or no side effects. For instance, the antimalarial drugs artemisinin and quinine are extracted from *Artemisia annua* and *Cinchona* bark [1,2]. Antimicrobial drugs such as berberine, a natural pentacyclic isoquinoline alkaloid, are derived from the stems and roots of *Berberis species* [3]. Natural products have been proven to be excellent and reliable sources for the development of new drugs.

Chronic kidney disease (CKD) has been recognized as a major and increasing health problem worldwide. The global estimated prevalence of CKD is between 8% and 16% [4,5]. In recent decades, significant progress has been made to gain insights into the treatments and consequences of CKD around the globe [6]. CKD is a progressive systemic disease, which changes the function and structure of the kidneys irreversibly over months or years. The current diagnosis of CKD relies on a chronic reduction in renal function and structural kidney damage. The international guidelines define CKD by a glomerular filtration rate (GFR) of less than 60 mL/min/1.73 m², albuminuria of at least 30 mg per 24 h, or markers of kidney damage persisting for at least 3 months [7]. Diabetes, hypertension, and obesity are important contributors to the global burden of disease and are the most common traditional risk factors for CKD [8]. Other causes such as glomerulonephritis, infection, and environmental exposures are common in many developing countries [4]. CKD is associated with risks of adverse clinical outcomes, such as cardiovascular disease, end-stage renal disease (ESRD), and increased mortality [9,10,11]. Thus, it is critical to find agents that can be used to effectively prevent and treat CKD.

Various functional and bioactive compounds from natural products have been identified as having critical properties in the treatment of CKD [12]. Among these, polyphenolic compounds exert multiple biological properties [13,14,15]. Flavonoids, a class of polyphenolic compounds, are characterized by a C6–C3–C6 backbone structure and are the most indispensable components presented in the human diet [16,17,18]. Flavonoids are well known for their beneficial effects on health and many biological functions, including antioxidative [19,20], antimicrobial [21,22], cardioprotective [23,24], and anticancer activities [25,26,27]. Specific flavonoids and a series of plant extracts containing flavonoids have been employed in cell or animal models of kidney disease for different types of investigations [28]. The results showed that flavonoids may have preventive effects in vitro or in vivo and provided a potential treatment for the disease. This paper systematically reviewed the functions and beneficial effects of flavonoids in CKD.

## 2. Diagnosis and Staging of CKD

Many people are asymptomatic in early-stage CKD and identified by chance through routine screening tests with serum chemistry profiles and urine studies. However, depending on the cause of CKD, some people have symptoms directly as a result of impaired kidney function. CKD is characterized by a reduction in nitrogenous waste excretion and many uremic retention solutes called uremic toxins accumulated in the body. Many of those uremic toxins contribute to inflammation, cardiovascular disease, immune dysfunction, platelet dysfunction, and increased bleeding risk, as well as CKD progression [29,30].

Chronic kidney disease is defined as an abnormality in kidney structure or function presenting for more than 3 months [31]. GFR is a measure of kidney function. The urine albumin-to-creatinine ratio (ACR) is a kidney damage marker [7]. The diagnosis includes one or more of the following: (1) GFR less than 60 mL/min/1.73 m^2^; (2) albuminuria (i.e., urine albumin ≥30 mg/24 h or ACR ≥30 mg/g); (3) abnormalities in urine sediment; (4) abnormalities detected by histology or structure damage detected by imaging; (5) abnormalities owing to tubular disorders; or (6) history of kidney transplantation. CKD has five stages classified by the CGA classification (cause, GFR category, and albuminuria category) [7]. Once CKD is diagnosed, the next step is to determine the staging, as shown in Table 1 [7,32].

## 3. Pathophysiology of CKD

There are two mechanisms for the occurrence of chronic kidney disease: an initial trigger and a perpetuating mechanism [33]. Initial stimulation may be caused by inflammation, or immune-mediated or toxic injury. This process leads to over-filtration and hypertrophy of the nephrons, resulting in changes in the glomerular structure and podocytes, which can damage the filtration system. Ultimately, these persistent injuries lead to nephrosclerosis and a further decline in renal function. The final common pathological manifestation of chronic kidney disease is renal fibrosis and is characterized by glomerulosclerosis, tubular atrophy, and interstitial fibrosis [34].

Global glomerulosclerosis is caused by injury and dysfunction of podocytes and endothelial cells, and the proliferation of smooth muscle cells and mesangial cells [35,36,37,38]. Glomerular microinflammation activates mesangial cells to proliferate and secrete several types of inflammatory cytokines, chemokines, and adhesion molecules, and to produce an excessive extracellular matrix (ECM), all of which participate in the process of glomerulosclerosis [39]. Podocyte loss results in local bulging of the glomerular basement membrane (GBM) when glomerular pressures increase, which leaves the GBM to form a synechia attachment with Bowman’s capsule, thus contributing to the first ‘committed step’ of glomerulosclerosis [40,41]. Tubular epithelial cells release various bioactive molecules including reactive oxygen species (ROS) and pro-inflammatory cytokines and chemokines to favor the recruitment of inflammatory cells, the activation of fibroblasts, and the loss of endothelial cells, which eventually lead to tubular atrophy, tubulointerstitial inflammation, and fibrosis [42,43].

Emerging evidence suggests that oxidative stress and inflammation, as well as their interaction, play pivotal roles in the pathogenesis and progression of CKD [44,45]. Inflammation has a prominent role in initiating renal fibrosis. Together with the activation of resident kidney immune cells, leukocytes and fibrogenic cells including T cells, B cells, monocytes/macrophages, dendritic cells, and mast cells are recruited to the glomerulus and renal interstitium, which leads to increase production of pro-inflammatory cytokines [46,47,48]. Activated leukocytes generate ROS, chlorine, and nitrogen species, thus aggravating and perpetuating oxidative stress [44,45]. With the activation of matrix-producing cells and the release of profibrotic cytokines, an excessive ECM is accumulated, which results in the renal structure and function gradually disappearing.

## 4. Flavonoids

### 4.1. Structure and Classification of Flavonoids

**Figure 1:**
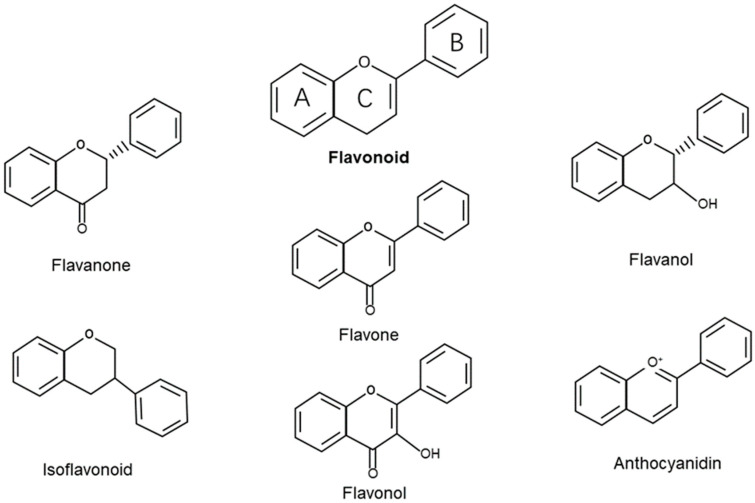
Chemical structures and classification of flavonoids.

Natural compounds constitute promising candidates in the therapy of various diseases. Among others, flavonoids stand out for being widely distributed in fruits, vegetables, grains, herbs, and beverages [49]. There are now more than 8000 varieties of flavonoids that have been structurally identified with a wide variety of biological properties [50]. Flavonoids are polyphenolic compounds synthesized in plants and as bioactive secondary metabolites. Structurally, flavonoids have a well-known chemical structure characterized by 15 carbon atoms (C6-C3-C6) that are arranged to form two benzene rings named A and B. The A-ring and B-ring are linked through a three-carbon bridge that usually arises as an oxygenated heterocyclic ring named C [51] (Figure 1). Based on the degree of saturation and the level of oxidation of the C-ring, and different connections between the B-ring and C-ring, flavonoids can be classified into different groups, such as flavones (e.g., apigenin, rutin, and luteolin), flavonols (e.g., quercetin, kaempferol, myricetin, and fisetin), flavanol (e.g., epigallocathechin), isoflavonoids (e.g., genistein and daidzein), flavanones (e.g., naringin, naringenin, and hesperidin), and anthocyanidins (e.g., apigenidin, cyanidin, and malvidin) [18,50]. In recent years, flavonoids have attracted people’s attention. Epidemiological and experimental studies have pointed to the health benefits associated with flavonoid intake [52]. Flavonoids are effective antioxidants that can protect plants from adverse environmental conditions [53]. Therefore, flavonoids have been evaluated for possible beneficial effects on a variety of acute and chronic human diseases. In vitro and in vivo studies have shown that flavonoids exert numerous benefits, such as anti-inflammatory [54,55], antioxidant [56,57], anticardiovascular [58], neuroprotective [59,60], and strong anticancer effects [61,62,63].

### 4.2. Metabolism of Flavonoid

The diversity of flavonoid structures undoubtedly contributes to the highly variable bioavailability between individuals. After absorption, flavonoids are widely metabolized in the gastrointestinal microbial and liver metabolism. Most dietary flavonoids in nature exist in aglycone form or are bound to glycosides. Only a few glucosides can be absorbed in the proximal intestine. A large proportion of unabsorbed flavonoids reach the colon where they are exposed to microbiome-mediated hydrolysis, fermentation, and catabolism into smaller molecules such as phenolic and aromatic acids, which may become bioavailable [64]. The metabolites of flavonoids are transported to the liver via the portal vein through the epithelium. Flavonoids undergo intrahepatic metabolisms such as methylation, sulfation, or glucuronidation before being released into the circulation and tissue uptake [64]. However, it is still unclear how tissues uptake flavonoid metabolites and how they are subsequently distributed. The gut microbiome plays a critical role in flavonoid metabolism. In addition, food composition, such as fat and protein intake, age, sex, and genotype may also affect flavonoids’ metabolic processes and bioavailability [64,65,66]. The efflux of flavonoids from the body is via the kidney, intestinal epithelium, and bile excretion [64]. Furthermore, to improve the low biological activity of flavonoids, various processes have been employed to optimize their absorption and bioavailability by using different delivery systems and absorption enhancers, changing the absorption site and metabolic stability [67].

## 5. Bioactivities of Flavonoids in CKD

### 5.1. Antidiabetic Effect

Diabetes mellitus (DM) is one of the prevailing global health problems throughout the world. It is a metabolic disorder characterized by an elevation in blood glucose due to insufficient or inefficient insulin [68]. All cells are chronically exposed to high plasma glucose levels and some manifest progressive dysfunction. The kidney is the most important target of microvascular damage in diabetes. Many flavonoids are reported to improve hyperglycemia and increase insulin sensitivity in in vitro and in vivo studies [69,70].

Flavonoids can interact with several molecular pathways to intervene in glucose metabolism, which is involved in glucose uptake by tissues, insulin sensitivity and secretion from β-cells, and the inhibition of intestinal glucose absorption [71]. The antidiabetic action of quercetin involves the reduction in lipid peroxidation, glucose absorption by glucose transporter type 4 (GLUT4), the inhibition of insulin-dependent activation of phosphoinositide 3-kinases (PI3K), stimulation of glucose uptake in muscle cells, and activation of AMP-activated protein kinase (AMPK) [72,73,74,75]. Quercetin and kaempferol could enhance insulin signaling transduction by inducing the phosphorylation of insulin receptor (IR) and insulin receptor substrate-1 (IRS-1) [76,77]. Kaempferol improved cell viability, decreased cell apoptosis, and promoted the secretion and synthesis of insulin in β-cells [78]. It could also activate the AMPK signaling pathway to increase glucose uptake [77]. Epigallocatechin gallate (EGCG) and genistein had a similar function in activating the PI3K/protein kinase B (AKT) pathway, increasing the phosphorylation of AMPK, and promoting GLUT4 translocation to improve glucose uptake [79,80]. Myricetin inhibited insulin secretion by restoring IR and IRS-1 and enhancing the phosphorylation of AKT and GLUT4 expression and translocation in high-fructose-fed rats [81,82]. Rutin reduced glucose absorption from the small intestine by inhibiting α-glucosidases and α-amylase involved in the digestion of carbohydrates [83]. Similar to other flavonoids, rutin stimulated tissue glucose uptake via insulin signaling, PI3K, and mitogen-activated protein kinase (MAPK) pathways [84]. Treatment with rutin also increased insulin levels by stimulating β-cells to produce insulin and showed antiapoptotic activities by increasing B-cell lymphoma 2 (Bcl-2) and decreasing the level of caspase-3 in streptozotocin (STZ)-induced diabetic rats [85]

### 5.2. Antihypertensive Effects

Hypertension is one of the leading risk factors of CKD that affects > 1 billion people worldwide. Nitric oxide (NO) from the endothelium plays a crucial role in regulating blood pressure (BP) [86]. A reduction in NO bioavailability and an elevation in the ROS level are key traits involved in endothelial dysfunction [87]. In addition, potassium and calcium channels are also important in NO-mediated vasodilation [88,89]. As hypertension persists, glomerulosclerosis occurs and, finally, causes atrophy and renal fibrosis. Efforts to improve endothelial dysfunction and increase NO bioavailability are of great significance in the treatment of hypertension.

The antihypertensive mechanisms of flavonoids mainly include (1) protection of endothelial cell function [90,91]; (2) suppression of the renin–angiotensin system [92,93]; (3) antioxidant stress and anti-inflammatory effects [94,95]; (4) inhibition of sympathetic excitation [96,97].

The antihypertensive effect of quercetin and kaempferol is due to their abilities to improve endothelial function and modulate the renin–angiotensin–aldosterone system (RAAS), and vascular smooth muscle cell contractility [92,98]. The ability of these compounds to improve endothelial dysfunction works through enhancing relaxation and suppressing contraction caused by endothelin-1 (ET-1) and increasing NO levels in plasma [99]. Quercetin also augmented NO through upregulating NO synthase activity in endothelial cells and enhanced vasodilation to attenuate hypertension via ameliorating oxidative stress [96]. EGCG and hesperetin could block voltage-operated Ca^2+^ channels and reduce ROS generation [100,101,102]. The hesperetin metabolite hesperetin-7-O-b-D-glucuronide (HPT7G) decreased BP by increasing the adhesion of NO synthase, reducing the levels of nitrous oxide, and enhancing endothelium-dependent vasodilation [103]. Hesperetin also suppressed hypertension by suppressing the RAAS and oxidant stress and blocking voltage-gated calcium channels [93,97,102]. Genistein exerted its antihypertensive effect by inhibiting the Ca^2+^-dependent non-receptor tyrosine kinase proline-rich tyrosine kinase 2 (PYK2) [104]. Luteolin ameliorated BP by signaling and activating the cyclic adenosine monophosphate (cAMP)/protein kinase cascade, which further activated NO synthase and increased the concentration of endothelial NO [105]. The ability of naringenin to reduce blood pressure was due to both membrane hyperpolarization and relaxation of vascular smooth muscles, which was affected by calcium-activated potassium channels [106]. Growing evidence suggests that flavonoid-rich foods in cardiovascular disease might lower BP by reducing sympathetic nervous system overactivity [96]. Vaccarin abrogated the increased plasma renin, angiotensin II, norepinephrine, and basal sympathetic activity [107].

### 5.3. Anti-Inflammatory Effects

Inflammation has been recognized as a complex biological process that occurs in response to harmful stimuli and is a major risk factor for various diseases. It is well known that acute inflammation has physiological functions of defense and healing, but when the inflammatory regulatory mechanism changes, this can lead to a long-term inflammatory process, thus disturbing the homeostasis [108]. The inflammatory response involves the recruitment of innate immune cells, which in turn produce pro-inflammatory cytokines and chemokines that attract lymphocytes to trigger tissue damage. During the inflammatory immune response, ROS, reactive nitrogen species, and different proteases are produced, all of which can contribute to chronic inflammation [109]. Chronic inflammation is involved in the development of certain diseases, such as asthma, cancer, cardiovascular disease, diabetes, and neurodegenerative diseases.

Flavonoids have been shown to exert anti-inflammatory properties through different mechanisms such as modulation of immune cells and inhibition of enzymes and transcription factors. Studies have reported that flavonoids have an impact on immune cell activation, maturation, and signaling transduction, which can inhibit the production and secretion of cytokines and chemokines. Quercetin has been shown to inhibit the maturation of dendritic cells by the downregulation of CD80, CD86, major histocompatibility complex II (MHC-II), interleukin-6 (IL-6), and interleukin-12 (IL-12) and reducing T cell allogeneic proliferation [110]. Flavonoids could decrease the release of pro-inflammatory cytokines from mast cells, eosinophils, and other immune cells [111,112]. Kaempferol attenuated tumor necrosis factor alpha (TNF-α)-induced expression of epithelial intercellular cell adhesion molecule-1 (ICAM-1) and eosinophil integrin β2, and monocyte chemotactic protein-1 (MCP-1) transcription, hindering eosinophil–epithelial interaction [113]. Flavonoids from wild blueberries also prevented monocyte adhesion to human umbilical vein endothelial cells in a TNF-α-mediated pro-inflammatory environment [114].

Inflammation depends on a group of protein kinases such as tyrosine kinase, phosphoinositol kinase, protein kinase C (PKC), and phosphatidylinositol kinase. The inhibition of those enzymes by different types of flavonoids has been reported [115,116,117]. Several studies have found that flavonoids can also impact arachidonic acid metabolism through enzymes such as phospholipase A2, cyclooxygenase, and lipoxygenase, thus inhibiting the biosynthesis of prostaglandins, thromboxanes, and leukotrienes [118,119,120,121]. Baicalein significantly reduced the expression of cyclooxygenase-2 (COX-2), prostaglandin E2 (PGE2), and lipoxygenase-1 (LOX-1) to promote a neuroprotective effect [122]. Flavonoids could also modulate protein kinases by the inhibition of transcription factors, such as nuclear factor kappa-B (NF-κB). NF-κB regulates several cytokines, chemokines, and cell adhesion molecules involved in inflammation [123]. For example, fisetin inhibited the renal expression of IL-6, interleukin 1 beta (IL-1β), TNF-α, and COX-2 to alleviate inflammation and apoptosis through inhibiting NF-κB p65 and MAPK signaling pathways [124].

### 5.4. Antioxidant Effects

The human body maintains homeostasis through maintaining the balance between oxidants and antioxidants through antioxidant defense systems. If the antioxidant defense is impaired, the production of ROS increases. ROS cause oxidative stress upon reacting with molecules such as lipids, proteins, or nucleic acids. Lipid peroxidation by ROS causes cellular membrane damage, which eventually causes cell death [57].

Flavonoids, which act as exogenous antioxidants by their ability to donate electrons to peroxynitrite, hydroxyl, and peroxyl radicals, have been proven to exhibit a noticeable positive influence in stabilizing the aforementioned radicals, reducing the levels of reactive oxygen and other free radicals in the human body [125]. Carbohydrate fragments from the structure of flavonoids play an important role in their antioxidant action. Aglycones have been proven to be stronger antioxidants than glycosides [126]. The antioxidant effect of flavonoids is achieved via direct and indirect mechanisms. The direct mechanism is eliminating reactive oxygen species directly [127]. Indirect antioxidant effects are related to stimulating the production or activation of antioxidant enzymes and suppressing pro-oxidant enzymes. Flavonoids have been found to activate intracellular antioxidant signaling pathways to accelerate the production of endogenous antioxidants such as glutathione (GSH), superoxide dismutase (SOD), and catalase (CAT), and inhibition of ROS-generating enzymes such as xanthine oxidase, myeloperoxidase (MDA), and nicotinamide adenine dinucleotide phosphate (NADPH) oxidase [128,129]. Meanwhile, flavonoids could chelate metal ions, thus reducing the formation of free radicals [130,131]. For example, quercetin could modulate NADPH oxidase-dependent oxidative stress under different pathological conditions [132,133,134]. Baicalin remarkably inhibited oxidative stress via suppressing MDA activity and enhancing SOD and GSH activity in rats [135]. Nuclear factor erythroid 2 (Nrf2) is a transcription factor responsible for regulating the production of endogenous antioxidants under oxidative stress [136]. Flavonoids such as quercetin, naringenin, baicalin, and genistein have been reported to exert a protective effect in various diseases through activation of the Nrf2 signaling pathway and diminish the spontaneous degradation of the Nrf2 protein [137,138,139,140]. Flavonoids competitively bind with the kelch-like ECH-associated protein 1 (KEAP1) protein in the Nrf2 binding site, resulting in Nrf2 protein translocation into the nucleus and activation of downstream proteins [141,142].

## 6. Flavonoids in CKD

### 6.1. Flavonoids in Diabetic Nephropathy

Diabetic nephropathy (DN), the most common complication in diabetes, leads to a deterioration of renal function and progression to ESRD [143]. DN is characterized by urine albumin excretion, diabetic glomerular lesions, and a reduction in GFR. Accumulating evidence has demonstrated that oxidative stress and inflammation prompted by hyperglycemia play paramount roles in the pathogenesis and progression of DN. Various studies have evaluated the role of flavonoids in DN, most of them reporting a positive effect on renal function (Table 2).

**Figure 2:**
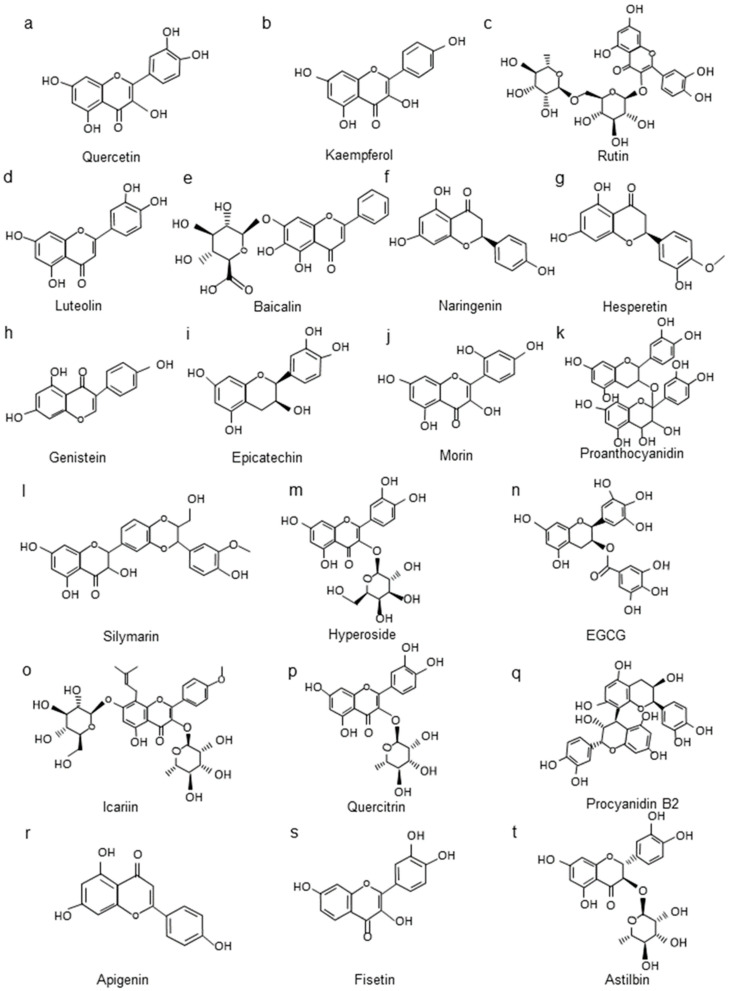
The chemical structures of the flavonoids discussed in CKD. (**a**–**t**): the flavonoid substances and their structural formulas mentioned in CKD.

Studies have revealed that quercetin (Figure 2a) could prevent glomerular and tubular damage in STZ-induced diabetic rats by reducing lipid peroxidation and increasing SOD and CAT activity [144]. In high-fat-diet/STZ-induced DN rats, quercetin could attenuate urine microalbumin excretion, the serum level of creatinine, hyperglycemia, and lipid metabolism disorders and mitigate renal histopathological lesions through suppressing the ROS and renal NOD-like receptor family, and the pyrin domain containing 3 (NLRP3) inflammasome [145]. It could also reduce free radicals by decreasing the levels of MDA, IL-1β, TNF-α, and advanced glycation end products (AGEs), and by increasing the activity of SOD and glutathione peroxidase (GSH-Px) [146]. Quercetin also improved renal function in rats with DN by inhibiting the overexpression of transforming growth factor beta 1 (TGF-β1) and connective tissue growth factor (CTGF) [147]. In db/db mice, quercetin effectively inhibited mesangial cell proliferation through reactivating the Hippo pathway [148].

Kaempferol (Figure 2b) significantly reduced renal inflammation, fibrosis, and kidney dysfunction in diabetic mice by regulating tumor necrosis factor receptor associated factor 6 (TRAF6) [149]. It also ameliorated renal injury and fibrosis by enhancing the release of glucagon-like peptide-1 (GLP-1) and insulin, and by inhibiting ras homolog family member A (RhoA)/Rho Kinase [150]. Kaempferol reduced renal inflammation, apoptosis, and the levels of ROS and MDA and stimulated SOD and GSH levels by the upregulation of the Nrf2/heme oxygenase-1 (HO-1) axis [151].

Rutin (Figure 2c) administration effectively protected the kidney through influencing matrix metalloproteinases (MMPs) and inhibiting oxidative stress and the TGF-β1/mothers against DPP homolog (Smad)/ECM and TGF-β1/CTGF/ECM signaling pathways in STZ-induced DN rats [152,153]. In alloxan-induced DN rats, rutin ameliorated renal fibrosis and metabolic acidosis via reducing the metabolic acidosis-related genes aquaporin 2 (AQP2), aquaporin 3(AQP3), and arginine vasopressin receptor 2 (V2R) [154]. Another study in the same model showed that rutin combined with ramipril downregulated TGF-β1 and endoplasmic reticulum stress markers glucose-regulated protein 78 (GRP78) and C/EBP-homologous protein (CHOP) [155].

Luteolin (Figure 2d) might ameliorate glomerulosclerosis and interstitial fibrosis in db/db mice models by inhibiting the inflammatory response and oxidative stress through repressing signal transducer and activator of transcription 3 (STAT3) activation [156]. Luteolin might protect the filtration function of the basement membrane by upregulating podocin protein expression and delaying the apoptosis, deletion, and fusion of podocytes under high-glucose conditions [157]. Luteolin could also increase SOD activity and HO-1 protein and decrease the MDA content to exert an antioxidant effect in diabetic nephropathy [158].

**Table 2:** Flavonoids in diabetic nephropathy.

Animal Models	Flavonoids	Functions	References
STZ-induced DN rats	Quercetin	Increasing SOD and CAT activity; suppressing ROS and the NLRP3 inflammasome; scavenging free radicals; inhibiting TGF-β1 and CTGF	[144,145,146,147]
	Kaempferol	Upregulating the Nrf2/HO-1 axis	[151]
	Baicalin	Downregulating PI3K/Akt/mTOR signaling	[159]
	Rutin	Inhibiting TGF-β1/Smad/ECM and TGF-β1/CTGF/ECM signaling pathways; influencing MMPs	[152,153]
	Luteolin	Upregulating Nphs2; increasing SOD/HO-1 and decreasing MDA	[157,158]
	Naringenin	Downregulating TGF-β1 and IL-1β; downregulating ER stress markers ATF4, p-PERK, p-eIF2α, and XBP1s	[160,161]
	Hesperidin	Restoring the α-Klotho/FGF-23 pathway; activating the Nrf2/ARE pathway	[162,163]
STZ-induced DN mice	Kaempferol	Regulating TRAF6; inhibiting RhoA/Rho Kinase	[149,150]
	Baicalin	Restoring Klotho expression and inhibiting Klotho hypermethylation	[164]
	Genistein	Reducing phospho-ERK/ERK ratio	[165]
Alloxan-induced DN rats	Rutin	Regulating AQP2/AQP3/V2R genes; downregulating TGF-β1, GRP78, and CHOP	[154,155]
db/db mice	Quercetin	Reactivating the Hippo pathway	[148]
	Baicalin	Activating Nrf2 and inhibiting the MAPK-mediated inflammatory signaling pathway	[166]
	Luteolin	Repressing STAT3 activation	[156]

Baicalin (Figure 2e) ameliorated diabetic conditions in db/db mice by alleviating oxidative stress and inflammation, and its underlying mechanisms were associated with the activation of the Nrf2-mediated antioxidant signaling pathway and the inhibition of the MAPK-mediated inflammatory signaling pathway [166]. Baicalin protected podocytes by downregulating the activity of the PI3K/AKT/mammalian target of rapamycin (mTOR) signaling pathway in STZ-induced DN rats [159]. Baicalin could also alleviate renal injury in STZ-induced DN mice through restoring Klotho expression and inhibiting Klotho hypermethylation to counter TGF-β1 signaling [164].

In summary, flavonoids have been found to counter the adverse renal effects in mice or rats with STZ-induced diabetes, db/db mice, and alloxan-induced DN rats. Those flavonoids regulate DN in several ways, including exerting antioxidative stress and anti-inflammatory effects. Besides the aforementioned flavonoids, other common flavonoids such as naringenin [160,161], hesperidin [162,163], and genistein [165] (Figure 2f–h) have also been proven to exert protective effects in DN rat or mouse models through inhibiting the oxidative stress pathway and pro-inflammatory factors.

### 6.2. Flavonoids in Hypertensive Nephropathy

Hypertensive nephropathy (HN) is the second leading cause of CKD after diabetic nephropathy. Statistics suggest that 84% of adults with CKD and half of patients with DM sustained hypertension [167]. Hypertension usually lasts for >10 years, and the early clinical manifestation is nocturia which appears later than the pathological changes. The kidneys are usually already severely damaged when renal function abnormalities are discovered. High BP can impact each renal compartment: glomeruli, tubulointerstitium, and vessels [168]. This disease is usually preceded by distal tubular dysfunction, followed by glomerular dysfunction [169]. Renal tubules and glomerular filtration membranes will be damaged in high-pressure and hyperfiltration conditions, which can lead to structural changes in renal arterioles and hypertrophy and proliferation of smooth muscle cells [170]. With the pathological condition continuing, the renal arteriole walls are thickened, the lumens are narrowed, the renal plasma flow further reduces, and the renal function is damaged. The glomeruli also change from hypertrophy to focal segmental sclerosis [171].

The main treatment of hypertensive nephropathy is to effectively reduce blood pressure. However, besides the antihypertensive effect, flavonoids can also act directly on the kidneys to improve the development of renal injury (Table 3). In 2002, quercetin was demonstrated to inhibit the development of hypertension induced in rats by chronic inhibition of NO synthesis with L-N G-Nitro arginine methyl ester (L-NAME). Meanwhile, quercetin reduced renal hypertrophy, proteinuria, renal parenchyma, and vascular lesions [172]. Quercetin has also been reported to significantly reduce the plasma creatinine concentration and prevent vascular dysfunction in deoxycorticosterone acetate (DOCA)-salt rats through restoring total GSH levels and improving renal glutathione S-transferase (GST) activity to maintain the antioxidant system [173,174]. The antioxidant effects of quercetin have also been shown in the treatment of renovascular hypertensive rats. Quercetin regulated hypertension and proteinuria and improved endothelium-dependent function through diminishing vascular production of the vasoconstrictor prostanoid thromboxane A2 (TXA2) [175]. A high dose of epicatechin (Figure 2i) and red wine polyphenols prevented the increase in systolic blood pressure, proteinuria, and endothelial dysfunction induced by DOCA-salt. Both can reduce NADPH oxidase activity and ET-1 levels, while epicatechin could also increase the transcription of Nrf2 [176,177]. Oral administration of morin (Figure 2j) reduced the raised plasma urea, uric acid, and creatinine levels in DOCA-salt rats [178]. The administration of rutin significantly attenuated the blood pressure along with a decrease in the plasma renin content and tissue thiobarbituric acid reactive substances (TBARS) and an increase in GSH levels in two-kidney, one-clip rat (2K1C) models [179]. Grape seed proanthocyanidins (GSPE) (Figure 2k) have been reported to be antioxidant and free radical scavengers, which can improve proteinuria, renal hypertrophy, and renal fibrosis through suppressed c-Jun N-terminal kinase (JNK) and p38 kinase pathways in DOCA-salt rats [180]. GSPE also significantly reduced albuminuria, inflammatory cell infiltration, and MCP-1 and IL-1β accumulation in the kidneys of spontaneously hypertensive rats (SHRs) [181]. In fructose-fed hypertensive rats, genistein administration led to endothelial nitric oxide synthase (eNOS) activation and NO synthesis in the kidney, restored angiotensin-converting enzyme and PKC-βII, and preserved renal ultrastructural integrity [182].

The prevention or amelioration of renal injury in HN by flavonoids is, in part, related to their function in preventing hypertension. Meanwhile, flavonoids can also interfere directly with the renal parenchyma through various mechanisms of antioxidative stress or anti-inflammation to prevent the development of renal injury.

### 6.3. Flavonoids in Glomerulonephritis

Glomerulonephritis (GN) is a heterogeneous group of diseases, accounts for about 20% of CKD cases in most countries, and frequently affects young people, which is likely to progress to ESRD [183]. The clinical presentation of glomerulonephritis is variable, including hypertension, proteinuria, hematuria, and raised serum creatinine concentrations. The most common glomerulonephritis types are IgA nephropathy, membranous glomerulonephritis, minimal change disease, focal segmental glomerulosclerosis (FSGS), membranoproliferative glomerulonephritis, and rare complement-associated types of glomerulonephritis such as dense deposit disease and C3 glomerulonephritis [183]. To date, a limited number of studies have focused on flavonoids in glomerulonephritis (Table 4).

Baicalin suppressed Notch1-Snail pathway activation in podocytes and alleviated glomerulus structural disruption and dysfunction in adriamycin (ADR)-induced nephropathy [184]. Total flavonoids in Astragali Radix (AR) were reported to protect against ADR-induced nephropathy related to the protection of renal filtration function and regulation of blood pressure, which might involve the regulation of the immune system and RAAS [185]. Silymarin (Figure 2l) was shown to decrease plasma creatinine and urea levels and normalize renal histopathology by suppressing renal MDA and GSH depletion [186]. Hyperoside (Figure 2m) could inhibit ADR-induced mitochondrial dysfunction and podocyte injury through regulating mitochondrial fission by restoring the expression of mitofusin 1 (MFN-1) [187]. EGCG (Figure 2n) was shown to significantly decrease glomerular and tubulointerstitial injury in immune-mediated glomerulonephritis by inhibiting MAPK pathways and phosphorylation of extracellular signal-regulated kinase (ERK)1/2 [188]. EGCG attenuated FSGS through the suppression of oxidant stress and cell apoptosis by inhibiting the hypoxia inducible factor 1 subunit alpha (HIF-1α)/angiopoietin like 4 (ANGPTL4) pathway [189]. Icariin (Figure 2o) treatment ameliorated renal damage in IgAN rats by the inactivation of the NF-κB pathway and the NLRP3 inflammasome [190].

**Table 4:** Flavonoids in glomerulonephritis.

Animal Models	Flavonoids	Functions	References
Adriamycin-induced rat nephropathy	Baicalin	Suppressing the Notch1-Snail pathway	[184]
	Total flavonoids in Astragali Radix	Regulating the immune system and RAAS	[185]
	Silymarin	Suppressing renal MDA and GSH depletion	[186]
Adriamycin-induced mouse nephropathy	Hyperoside	Regulating mitochondrial fission by restoring the expression of Mfn-1	[187]
	Epigallocatechin-3- gallate	Suppressing oxidant stress and cell apoptosis; inhibiting the HIF-1α/ANGPTL4 pathway	[189]
Anti-GBM-GN in 129/svJ mice	Epigallocatechin-3- gallate	Inhibiting MAPK pathways and phosphorylation of ERK1/2, JNK, and p38	[188]
Bovine gamma-globulin-induced rat IgA nephropathy	Icariin	Inhibiting the NF-κB pathway and mediating NLRP3 inflammasome activation	[190]

### 6.4. Flavonoids in Lupus Nephritis

Lupus nephritis (LN) is the most serious complication of systemic lupus erythematosus (SLE) and a major cause of mortality and morbidity in SLE patients [191]. Approximately 25–50% of SLE patients suffer from LN, which is characterized by a high expression of inflammatory cytokines, glomerulonephritis, and impaired renal function. Immune complex deposition, inflammatory cell infiltration, and complement activation are the key features of LN [192]. Proteinuria is one of the major clinical manifestations of LN. Podocytes play a crucial role in glomerular filtration and renal function preservation [193]. Excessive mesangial cell proliferation can affect podocyte function and is the main pathological characteristic of LN. Inhibition of mesangial cell proliferation can effectively aggravate renal damage [194]. A large proportion of patients with LN eventually progress to ESRD. Therefore, it is urgent to elucidate the underlying mechanisms of LN and develop effective drugs for LN therapy.

Flavonoids have shown markedly protective effects in LN (Table 5). Baicalin could become a promising therapeutic medicine for the treatment of SLE. It has been shown to decrease the levels of ROS and NF-κB phosphorylation with induction of Nrf2/HO-1 signaling and suppression of the NLRP3 inflammasome, which attenuated proteinuria and impaired renal function and histopathology in lupus mice [195]. Baicalin could also inhibit mTOR activation and Tfh cell differentiation while promoting Foxp3+ regulatory T cell differentiation in LN [196]. Naringenin decreased the levels of anti-nuclear and anti-dsDNA autoantibodies while increasing the percentage of Treg cells and preventing kidney damage and fibrosis of LN [197]. Icariin reduced the serum anti-dsDNA antibody level and immune complex deposition by suppressing the NLRP3 inflammasome, NF-κB activation, and TNF-α and C-C motif chemokine ligand 2 (CCL2) production in MRL/lpr mice [198]. Quercetin was observed to improve podocin, proteinuria levels, and the renal ultrastructure. It also inhibited the tissue expression of IL-6, TNF-α, TGF-β1, Bcl2 associated X (Bax), and TBARS while significantly increasing CAT and SOD expressions in the pristane-induced LN mouse model [199]. In the chronic graft-versus-host disease (cGVHD) mouse model, quercitrin (Figure 2p) ameliorated the symptoms of lupus nephritis due to the inhibition of CD4 + T cell activation and anti-inflammatory effects on macrophages [200]. Procyanidin B2 (Figure 2q) significantly reduced renal immune complex deposition and serum anti-dsDNA levels and inhibited NLRP3 inflammasome activation in MRL/lpr mice [201]. Apigenin (Figure 2r) inhibited the expression of NF-κB-regulated antiapoptotic molecules to promote the apoptosis of lupus antigen-presenting cells (APCs) and Th1, Th17, and B cells in the lupus mouse model [202]. In the same LN model, EGCG promoted the Nrf2 antioxidant signaling pathway while inhibiting NLRP3 inflammasome activation in the kidney [203]. Fisetin (Figure 2s) reduced the expression of pro-inflammatory cytokines IL-6, TNF-α, and IL-1β and chemokines C-X-C motif chemokine ligand 1 (CXCL-1), C-X-C motif chemokine ligand 2 (CXCL-2), and C-C motif chemokine ligand 3 (CCL3). Furthermore, the elevated level of Th17 cells in the pristane-induced LN mouse model was disrupted by fisetin [204]. Astilbin (Figure 2t) could also mitigate the development of glomerulonephritis in MRL/lpr mice by decreasing multiple cytokines and functional activated T and B cells [205].

## 7. Prospects and Conclusions

**Figure 3:**
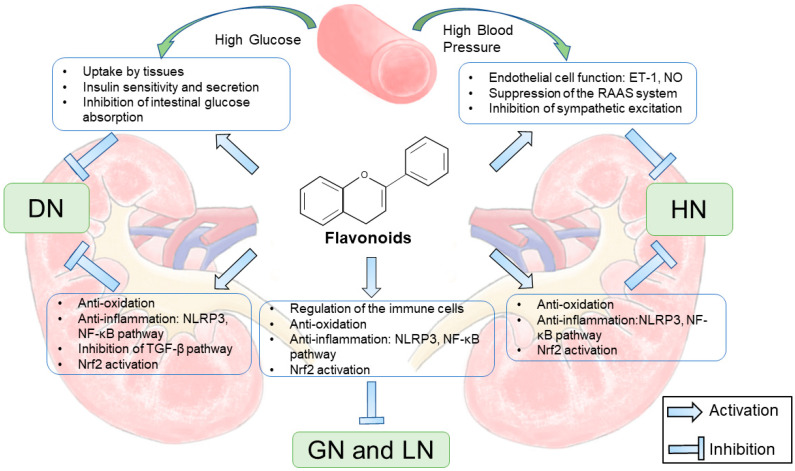
The nephroprotective mechanisms of flavonoids in CKD.

CKD is a public health epidemic associated with an increased risk of death. Flavonoids are groups of various compounds found naturally in many plants and fruits and have been reported to possess a wide range of health benefits. This review of recent progress in the role and mechanisms of action of flavonoids in CKD shows that flavonoids can attenuate kidney injury both directly and indirectly (Figure 3). Flavonoids exert significant biological activities in CKD, such as antidiabetic, anti-inflammatory, antihypertensive, and antioxidant effects, and alleviate renal fibrosis. These data support a role of flavonoids as potential compounds for further studies to develop new therapeutic agents for CKD. However, few clinical studies have been carried out, which indicates that the clinical application of flavonoids needs further research. In addition, it is important to determine the metabolites produced after the administration and improve the bioavailability, which may also contribute to better effects of flavonoids.

## Figures and Tables

**Table 1:** Staging of chronic kidney disease.

GFR Category	
G1	≥90 mL/min/1.73 m^2^
G2	60–89 mL/min/1.73 m^2^
G3a	45–59 mL/min/1.73 m^2^
G3b	30–44 mL/min/1.73 m^2^
G4	15–29 mL/min/1.73 m^2^
G5	<15 mL/min/1.73 m^2^
**Albuminuria Category**	
A1	ACR < 30 mg/g
A2	ACR 30–300 mg/g
A3	ACR > 300 mg/g

**Table 3:** Flavonoids in hypertensive nephropathy.

Animal Models	Flavonoids	Functions	References
DOCA-salt rats	Quercetin	Restoring total GSH levels and reducing TBARS level; restoring MDA content and SOD expression and improving potassium depletion	[173,174]
	Epicatechin	Reducing NADPH oxidase activity and ET-1 levels; increasing Nrf2	[176]
	Red wine polyphenols	Reducing NADPH oxidase activity and ET-1 levels	[177]
	Grape seed proanthocyanidins	Suppressing the JNK/p38 kinase pathway	[180]
	Morin	Reducing plasma urea, uric acid, and creatinine levels	[178]
2K1C rats	Quercetin	Restoring total GSH content and reducing the vasoconstrictor TXA2	[175]
	Rutin	Decreasing tissue TBARS and increasing GSH levels	[179]
L-NAME rats	Quercetin	Reducing renal hypertrophy, proteinuria, renal parenchyma, and vascular lesions	[172]
SHRs	Grape seed proanthocyanidins	Upregulating cofilin1 and inhibiting the NF-κB pathway	[181]
Fructose-fed hypertensive rats	Genistein	Inhibiting ACE and PKC-βII and activating eNOS and NO synthesis	[182]

**Table 5:** Flavonoids in lupus nephritis.

Animal Models	Flavonoids	Functions	References
Pristane-induced lupus mice	Baicalin	Inducing Nrf2/HO-1 signal and NLRP3 expression	[195]
	Fisetin	Reducing Th17 cells; inhibiting the CXCL signaling pathway	[204]
	Quercetin	Increasing CAT and SOD1 expressions; lowering IL-6, TNF-α, TGF-β1, Bax, and TBARS	[199]
Lupus-prone MRL/lpr mice	Baicalin	Inhibiting mTOR activation; reducing mTOR-mediated Tfh cell expansion; increasing Tfr cells	[196]
	Naringenin	Decreasing anti-nuclear and anti-dsDNA autoantibodies; increasing the percentage of Treg cells	[197]
	Icariin	Suppressing the NLRP3 inflammasome and the NF-κB signaling pathway	[198]
	Procyanidin B2	Inhibiting IL-1β, IL-18, and NLRP3 inflammasome	[201]
	Astilbin	Decreasing functional activated T and B cells	[205]
Lupus-prone SNF1 mice	Epigallocatechin-3- gallate	Enhancing the Nrf2 antioxidant pathway and inhibiting the NLRP3 inflammasome	[203]
	Apigenin	Inhibiting autoantigen-presenting and stimulatory functions of APCs; causing apoptosis of hyperactive lupus APCs and T and B cells	[202]
Chronic GVHD mouse model	Quercitrin	Inhibiting CD4 + T cell activation	[200]

## Abbreviations

CKD: chronic kidney disease; GFR: glomerular filtration rate; ESRD: end-stage renal disease; ACR: albumin-to-creatinine ratio; ECM: extracellular matrix; GBM: glomerular basement membrane; DM: diabetes mellitus; DN: diabetic nephropathy; HN: hypertensive nephropathy; GN: glomerulonephritis; LN: lupus nephritis; GLUT-4: glucose transporter type 4; PI3K: phosphoinositide 3-kinases; AMPK: 5′-AMP-activated protein kinase; IR: insulin receptor; IRS-1: insulin receptor substrate-1; EGCG: epigallocatechin gallate; AKT: protein kinase B; MAPK: mitogen-activated protein kinase; Bcl-2: B-cell lymphoma 2; STZ: streptozotocin; NO: nitric oxide; ROS: reactive oxygen species; RAAS: renin–angiotensin–aldosterone system; ET-1: endothelin-1; PYK2: proline-rich tyrosine kinase 2; cAMP: cyclic adenosine monophosphate; MHC-II: major histocompatibility complex II; IL-6: interleukin-6; IL-12: interleukin-12; TNF-α: tumor necrosis factor alpha: ICAM-1: intercellular cell adhesion molecule-1; MCP-1: monocyte chemotactic protein-1; PKC: protein kinase C; COX-2: cyclooxygenase-2; PGE2: prostaglandin E2; LOX-1: lipoxygenase-1; NF-κB: nuclear factor kappa B; IL-1β: interleukin-1 beta; GSH: glutathione; SOD: superoxide dismutase; CAT: catalase; MDA: methane dicarboxylic aldehyde; NADPH: nicotinamide adenine dinucleotide phosphate; Nrf2: Nuclear factor erythroid 2; KEAP1: kelch-like ECH-associated protein 1; NLRP3: NOD-like receptor family, pyrin domain containing 3; AGEs: advanced glycation end products; GSH-Px: glutathione peroxidase; TGF-β1: transforming growth factor beta 1; CTGF: connective tissue growth factor; TRAF6: tumor necrosis factor receptor associated factor 6; HO-1: heme oxygenase-1; GLP-1: glucagon-like peptide-1; RhoA: ras homolog family member A; MMPs: matrix metalloproteinases; Smad: mothers against DPP homolog; AQP2: aquaporin 2; AQP3: aquaporin 3; V2R: arginine vasopressin receptor 2; GRP78: glucose-regulated protein 78; CHOP: C/EBP-homologous protein; STAT3: signal transducer and activator of transcription 3; mTOR: mammalian target of rapamycin; GST: glutathione S-transferase; TXA2: thromboxane A2; GSPE: grape seed proanthocyanidins; JNK: c-Jun N-terminal kinase; eNOS: endothelial nitric oxide synthase; MFN-1: mitofusin 1; ERK: extracellular signal-regulated kinase; HIF-1α: hypoxia inducible factor 1; ANGPTL4: angiopoietin like 4; CCL2: C-C motif chemokine ligand 2; Bax: Bcl2 associated X; TBARS: thiobarbituric acid reactive substances; APCs: antigen-presenting cells; CXCL-1: C-X-C motif chemokine ligand 1; CXCL-2: C-X-C motif chemokine ligand 2; CCL3: C-C motif chemokine ligand 3.
